# Association Between Spatial Heterogeneity Within Nonmetastatic Gastroesophageal Adenocarcinomas and Survival

**DOI:** 10.1001/jamanetworkopen.2020.3652

**Published:** 2020-04-27

**Authors:** Joseph Chao, Victoria Bedell, Jeeyun Lee, Min Sierra Li, Peiguo Chu, Yate-Ching Yuan, Dan Zhao, Samuel J. Klempner, Ren-Jang Lin

**Affiliations:** 1 Department of Medical Oncology and Therapeutics Research, City of Hope Comprehensive Cancer Center, Duarte, California; 2 Cytogenetics Core, City of Hope Comprehensive Cancer Center, Duarte, California; 3 Division of Hematology-Oncology, Department of Medicine, Samsung Medical Center, Sungkyunkwan University School of Medicine, Seoul, Korea; 4 Department of Biostatistics, City of Hope Comprehensive Cancer Center, Duarte, California; 5 Department of Pathology, City of Hope Comprehensive Cancer Center, Duarte, California; 6 Bioinformatics Core, City of Hope Comprehensive Cancer Center, Duarte, California; 7 Department of Medicine, Massachusetts General Hospital Cancer Center, Boston; 8 Harvard Medical School, Boston, Massachusetts; 9 Department of Molecular and Cellular Biology, Beckman Research Institute of City of Hope, Duarte, California

## Abstract

**Importance:**

Intratumoral heterogeneity has been recognized as a significant barrier in successfully developing targetable biomarkers for gastroesophageal adenocarcinoma (GEA) and may affect neoadjuvant precision medicine approaches.

**Objective:**

To describe intratumoral spatial heterogeneity of tumor cell populations in nonmetastatic GEA and its association with survival.

**Design, Setting, and Participants:**

This case series retrospectively identified 41 patients with GEA who underwent up-front surgical resection at a tertiary referral cancer center from January 1, 1989, through December 31, 2013. Survival was calculated from date of surgery to date of death through June 1, 2017. Data were analyzed from June 2, 2017, to March 1, 2019.

**Main Outcomes and Measures:**

Overall survival, intratumoral clonal composition determined by genomic single-nucleotide variation array and bioinformatic analysis, and intercellular tumoral distances determined by multiprobe fluorescence in situ hybridization.

**Results:**

Among the 41 patients included in the analysis (22 men [54%]; mean [SD] age, 63 [12] years), a high proportion (19 [46%]) presented with tumors possessing high intratumoral heterogeneity. Kaplan-Meier analysis demonstrated that cases with an intratumoral clonal composition count of at least 2 exhibited worse survival compared with cases with a clonal composition count of 0 to 1 (univariate hazard ratio, 3.92; 95% CI, 1.27-12.08; *P* = .02). This finding remained significant on multivariate analysis controlling for stage, Lauren histologic subtype, receipt of adjuvant therapy, and age (multivariate hazard ratio, 4.55; 95% CI, 1.09-19.04; *P* = .04). Multiprobe fluorescence in situ hybridization demonstrated intratumoral clonal populations coexisting at submillimeter distances with differing relevant oncogenic copy number alterations, such as *EGFR*, *JAK2*, *FGFR2*, *MET*, *CCND1*, *KRAS*, *MYC*, *PIK3CA*, *CD274*, and *PDCD1LG2*.

**Conclusions and Relevance:**

This study found that spatial intratumoral heterogeneity of oncogenic copy number alterations exists before metastatic dissemination, and increased heterogeneity was associated with worse outcomes in resected GEA. Baseline heterogeneity illustrates the challenges in GEA targeted therapy. Further study may offer insight into strategies on combinatorial and/or sequential targeted and immunotherapeutic approaches.

## Key Points

**Question:**

Is there an association between tumor cell heterogeneity at time of diagnosis in nonmetastatic gastroesophageal adenocarcinoma and prognosis?

**Findings:**

In this case series of 41 patients with gastroesophageal adenocarcinoma, a high degree of intratumoral heterogeneity was identified. The presence of clonal populations coexisting at submillimeter distances was associated with worse survival.

**Meaning:**

These findings suggest that understanding intratumoral heterogeneity is highly relevant for future precision medicine neoadjuvant strategies in gastroesophageal adenocarcinoma, and single-cell analytic approaches are recommended.

## Teaser

This case series describes intratumoral spatial heterogeneity of tumor cell populations and investigates its association with survival among patients with nonmetastatic gastroesophageal adenocarcinoma.

## Introduction

Precision medicine efforts in gastroesophageal adenocarcinoma (GEA) have been hampered by the recognition that intratumoral heterogeneity confounds results of genomic testing yielded from limited sampling of the tumor.^1^ Such intratumoral heterogeneity of actionable receptor tyrosine kinases, including *ERBB2*, *MET*, and *FGFR2*, affects targeted therapeutic strategies.^1,2,3,4^ Knowledge of intratumoral heterogeneity in gastric cancer at de novo nonmetastatic disease presentation remains limited, with recent studies providing emerging evidence.^1^ The Cancer Genome Atlas (TCGA) has laid the framework of copy number alterations (CNAs) predominating the mutational landscape of GEA, particularly in the chromosomal instability subtype of tumors.^5,6^ Pancancer TCGA studies have also deciphered intratumoral heterogeneity from whole-exome sequencing data, with worse survival observed in patients with higher heterogeneity.^7^ With this understanding, we undertook an in-depth study of GEA intratumoral heterogeneity and its association with survival using a genome-wide single-nucleotide variation (SNV; formerly termed single-nucleotide polymorphism) array panel. We focused on cases treated without neoadjuvant therapy before surgery to best capture de novo disease presentation and the molecular landscape that may affect neoadjuvant strategies incorporating targeted agents. We pursued multiprobe fluorescence in situ hybridization (FISH) analyses to discern at a single-cell level the presence and patterns of spatial genomic heterogeneity.

## Methods

### Study Population

For this case series, 41 patients with nonmetastatic GEA who underwent up-front surgical resection with curative intent were retrospectively identified from the City of Hope Biospecimen Repository. Waiver of consent was granted under a protocol approved by the institutional review board of City of Hope, as the proposed research presented no more than minimal risk of harm, and the waiver did not adversely affect the rights and welfare of the participants. Patients who underwent surgical resection from January 1, 1989, to December 31, 2013, without receipt of neoadjuvant chemotherapy, radiotherapy, and/or targeted therapy were selected to best capture innate heterogeneity at disease presentation. The explicit selection of patients to test the hypothesis that intratumoral heterogeneity at disease presentation is associated with survival follows published guidelines for case series studies.^8^

Available demographic, clinical, and pathologic characteristics were extracted from retrospective review of patients’ medical records and pathology reports. The staging system of the American Joint Committee on Cancer, 7th edition, was applied from pathology reports and used to define pathologic stage. Overall survival was calculated as time from the date of surgery to the date of death due to any cause through a cutoff date of June 1, 2017.

### SNV Array Panel Copy Number Analysis

For genomic tumor DNA isolation, hematoxylin-eosin–stained slides were evaluated by the pathologist (P.C.), and tumor areas were circled. Tumor tissue was microdissected from two 10-μm formalin-fixed, paraffin-embedded (FFPE) sections. The DNA was isolated using the FFPE plus low elution volume DNA isolation kit (Maxwell 16; Promega) and quantified with the manufacturer’s fluorometer (Quantus; Promega). The FFPE assay (OncoScan; ThermoFisher Scientific) was run according to the manufacturer’s directions using 80 ng of DNA. This molecular inversion probe technology enabled reliable analysis of 30-year-old FFPE specimens.^9^ The OSCHP files were analyzed with Nexus Express software, version 4.0 (Biodiscovery, Inc) and Chromosome Analysis Suite, version 3.1 (ThermoFisher Scientific). We assayed the CNAs of 891 cancer-related genes at 50- to 100-kilobase resolution using the Affymetrix OncoScan platform per the manufacturer’s instructions. Copy number gains were defined as CNAs of greater than 2 and 5 or less; amplifications, as CNAs of 6 or greater; shallow deletions, as CNAs of less than 2 and 1 or greater; and deep deletions, as CNAs of 0.

### Clonal Composition Analysis

Clonal composition was determined using a combination of 4 software tools: Chromosome Analysis, version 3.1 (ChAS; ThermoFisher Scientific), Nexus Express software for OncoScan (Biodiscovery, Inc), Oncoclone Composition,^10^ and Tuscanator, version 6 (provided by Sam Dougaparsad, PhD). Tuscanator script is derived from the published ASCAT (allele-specific copy number analysis of tumors) algorithm from Van Loo et al,^11^ whereas for a given aberrant segment, Tuscanator uses the mean smooth signal values (gaussian smoothed calibrated copy number estimate from the log2 ratio data) and the mean B-allele frequency (BAF) SNV values, with a range of copy numbers from 1 to 6. Briefly, Nexus Express was used to visualize a whole genome view and determine BAF patterns across each sample. OncoClone Composition was used to estimate the number of clones within a patient’s primary gastric tumor. OncoClone Composition is less predictive for high-ploidy samples; therefore, BAF and smooth signal average per segment were entered into Tuscanator software to generate a representative percentage of abnormal cells for each segment and subsequently determine clonal composition.

### Simultaneous and Sequential FISH Analyses

Copy number alterations were confirmed by FISH in select samples to confirm spatial heterogeneity signal patterns. The method used to pursue multiprobe FISH on FFPE has been previously described.^12^ Briefly, hematoxylin-eosin–stained slides were scanned on an image analyzer system (Duet; BioView). Fluorescent images were matched with hematoxylin-eosin staining and captured. For sequential hybridizations, the probe was removed by incubating in 70% formamide/2 × sodium saline citrate for 5 minutes at 75 °C. Images were matched with previous targets and captured. Two-dimensional spatial coordinates were noted for FISH images at micron resolution afforded by image-capturing software. Distances (d) between 2 coordinates (x_1_, y_1_) and (x_2_, y_2_) were calculated using the pythagorean theorem:

d = √(x_2_ – x_1_)^2^ + (y_2_ – y_1_)^2^

### Statistical Analysis

Data were analyzed from June 2, 2017, to March 1, 2019. All data were analyzed in R statistical software, version 3.4.3 (R Project for Statistical Computing). For continuous variables, Mann-Whitney tests were used. Continuous data are presented as mean (SD) unless otherwise stated, and 2-tailed *P* ≤ .05 was considered statistically significant. Overall survival was analyzed by Kaplan-Meier estimates, and hazard ratios were calculated from Cox proportional hazards regression models.

## Results

### Interpatient Tumoral Heterogeneity

Gastroesophageal adenocarcinoma samples from 41 patients (22 men [54%] and 19 women [46%]; mean [SD] age, 63 [12] years) were analyzed using the OncoScan platform. Thirty-seven samples were within quality control metric standards. Four samples failed the SNV quality control of normal diploid markers (ndSNVQC) metric. The allelic data for these samples was otherwise clear, and failure of ndSNVQC was likely owing to a lower number of normal diploid markers, high ploidy, or multiple clones in the sample. Four cases had normal copy number results. The percentage of abnormal cells in the remaining 37 cases ranged from 16% to 60%.

The Table lists the clinicopathologic characteristics of the study population and corresponding percentage of genomic changes, percentage loss of heterozygosity, and clonal composition count. Further analysis demonstrated a higher percentage of genomic changes associated strongly with Lauren intestinal subtype histology compared with diffuse subtype histology (median, 39.9% vs 4.2%; *P* = .001 by Mann-Whitney test) (eFigure 1A in the Supplement), and in tumors arising from the gastroesophageal junction, cardia, or proximal stomach vs tumors arising from the gastric body or antrum (median, 39.3% vs 14.4%; *P* = .01 by Mann-Whitney test) (eFigure 1B in the Supplement), consistent with TCGA trends. We had good representation of cases with TCGA chromosomal instability subtype with detection of multiple CNAs across the genome (eFigure 2 in the Supplement, top panel). In this case, multiple chromosomes also exhibited alternating disruption of BAFs, indicating both retention and loss of heterozygosity throughout the genome, pathognomonic for a chromothripsis event having occurred in this tumor’s evolution (eFigure 2 in the Supplement, bottom panel).

**Table.** Clinicopathologic Characteristics and Genomic Analyses Yielded From the Oncoscan Platform of 41 Gastric Cancer Cases

Patient No./sex/age at diagnosis, y	Year of surgery	Lauren classification	Location	Staging AJCC7	Received adjuvant therapy	LOH, %	Genome changed, %	Clonal composition count
1/M/60s	1989	Intestinal	GEJ	T3N3	No	20.30	48.30	2
2/M/60s	1989	Indeterminate	GEJ	T3N1	No	0.54	0.52	0
3/M/60s	1990	Mixed	GEJ/fundus	T2N1	NR	17.80	39.00	3
4/F/50s	1994	Intestinal	Stomach/distal body lesser curve	T3N0	No	2.29	49.60	2
5/M/70s	1994	Intestinal	GEJ	T3N0	No	12.00	73.00	3
6/F/30s	1994	Intestinal	Stomach/proximal	T4N2	Yes	1.01	18.10	2
7/M/60s	1995	Diffuse	GEJ/stomach/cardia	T3N2	No	1.49	20.30	1
8/M/70s	1995	Indeterminate	Stomach/cardia	T4aN3	No	5.10	34.40	2
9/F/70s	1996	Indeterminate	Stomach/antrum	T3N2	No	0.43	4.70	0
10/F/80s	1996	Indeterminate	GEJ	T3N3	No	2.05	13.30	1
11/F/70s	1996	Mixed	Stomach/antrum	T4aN3	No	1.99	12.60	2
12/F/50s	1997	Intestinal	GEJ	T3N2	Yes	0.72	27.70	2
13/F/50s	1997	Diffuse	Stomach/body (linitis)	T4aN3	Yes	0.86	0.69	1
14/M/70s	1999	Diffuse	Stomach/antrum	T4aN3	No	0.38	1.39	1
15/M/60s	2000	Mixed	Stomach/cardia	T2N1	Yes	7.43	2.71	1
16/M/70s	2001	Indeterminate	GEJ	T3N2	Yes	26.90	60.90	3
17/M/50s	2001	Intestinal	GEJ	T3N2	Yes	16.10	46.50	2
18/F/50s	2001	Diffuse	Stomach/body	T2N2	Yes	0.74	4.66	1
19/M/60s	2003	Intestinal	Stomach/distal lesser curve	T3N3	Yes	1.84	14.40	1
20/M/60s	2004	Indeterminate	GEJ	T3N3	No	13.40	66.30	3
21/M/70s	2004	Intestinal	Stomach/antrum	T3N0	NR	19.30	62.10	2
22/M/50s	2005	Diffuse	Stomach/lesser curvature	T3N1	Yes	0.11	0.24	0
23/F/30s	2005	Diffuse	Stomach/middle body greater curve	T3N1	Yes	2.80	17.60	1
24/M/80s	2006	Diffuse	Stomach/antrum	T3N0	No	14.90	44.30	1
25/M/70s	2008	Intestinal	Stomach/distal body	T3N1	Yes	30.10	63.10	3
26/F/30s	2008	Diffuse	Stomach/distal lesser curve	T3N1	Yes	14.40	34.50	1
27/M/60s	2008	Indeterminate	Stomach/distal lesser curve	T2N2	Yes	13.50	22.60	1
28/F/60s	2009	Indeterminate	Stomach/proximal greater curve	T3N1	No	24.70	68.80	2
29/F/50s	2009	Diffuse	GEJ	T3N0	Yes	14.30	51.20	3
30/M/50s	2010	Intestinal	GEJ	T2N1	Yes	4.99	41.30	3
31/F/40s	2010	Intestinal	Stomach/distal body	T3N2	Yes	41.00	48.80	4
32/M/50s	2011	Diffuse	Stomach/antrum	T2N0	Yes	0.44	0.15	0
33/F/70s	2012	Diffuse	Stomach/body	T1N3	Yes	0.72	31.00	1
34/F/70s	2012	Diffuse	Stomach/antrum	T3N1	Yes	0.83	3.18	0
35/M/60s	2012	Mixed	Stomach/antrum	T3N3	Yes	0.23	0.18	0
36/F/40s	2013	Diffuse	Stomach/body	T3N0	Yes	0.68	0.00	0
37/M/50s	2013	Intestinal	Stomach/cardia	T2N3	Yes	9.43	58.40	3
38/M/60s	2013	Diffuse	Stomach/middle distal body	T3N3	Yes	0.41	3.58	0
39/F/60s	2014	Intestinal	Stomach/distal body	T2N1	Yes	3.78	24.10	1
40/F/60s	2014	Diffuse	Stomach/antrum	T4aN2	Yes	2.92	46.00	2
41/F/60s	2014	Diffuse	Stomach/lesser curve (linitis)	T3N0	Yes	3.07	2.52	0

Abbreviations: AJCC7, American Joint Committee on Cancer *Cancer Staging Manual, Seventh Edition*, GEJ, gastroesophageal junction; LOH, loss of heterozygosity; NR, not recorded.

We observed interpatient tumoral heterogeneity across nearly all cases, with a variety of genomic alterations occurring in selected genes of interest as represented in the Oncoprint diagram (eFigure 3 in the Supplement).^13,14^ Multiple oncogenic CNAs were observed across patients, including high copy gains in *EGFR*, (OMIM 131550), *ERBB2* (OMIM 164870), *JAK2* (OMIM 147796), *FGFR2* (OMIM 176943), *MET* (OMIM 164860), *VEGFA* (OMIM 192240), *KRAS* (OMIM 190070), *NRAS* (OMIM 164790), *PIK3CA* (OMIM 171834), *CCNE1* (OMIM 123837), *CCND1* (OMIM 168461), *CDK4* (OMIM 123829), *CDK6* (OMIM 603368), *AURKA* (OMIM 603072), *MDM2* (OMIM 164785), *CD274* (OMIM 605402), and *PDCD1LG2* (OMIM 605723).

### Intratumoral Heterogeneity Determined by SNV Array Analyses

To further analyze the presence of intratumoral heterogeneity across our cohort, we calculated clonal composition using a combination of Chromosome Analysis, version 3.1, Nexus Express software for OncoScan, OncoClone Composition,^10^ and Tuscanator, version 6. We identified a clonal composition count of 4 in 1 case (2%), 3 in 8 cases (20%), and 2 in 10 cases (24%) (Table). Nine cases were classified as being composed of a single homogeneous cell population (clonal composition count of 0). Four of these 9 cases did not have any detectable copy number changes. Of the 5 remaining cases, 3 were notable for small genomic areas of focal DNA amplification, with the rest of the genome exhibiting normal copy number data. The remaining 2 cases had 16% abnormal cells, which is below the manufacturer’s limit of sensitivity of 20%; however, the percentage of abnormal cells was resolved using BAF and smooth signal averages. Thirteen samples had 1 clone identified ranging from 18% to 55% of the cells within the respective tumor sample. In total, intratumoral heterogeneity as represented by a clonal composition count greater than 1 was confirmed in 19 of 41 samples (46%).

### Association of Increased Heterogeneity With Shorter Overall Survival

**Figure 1.**
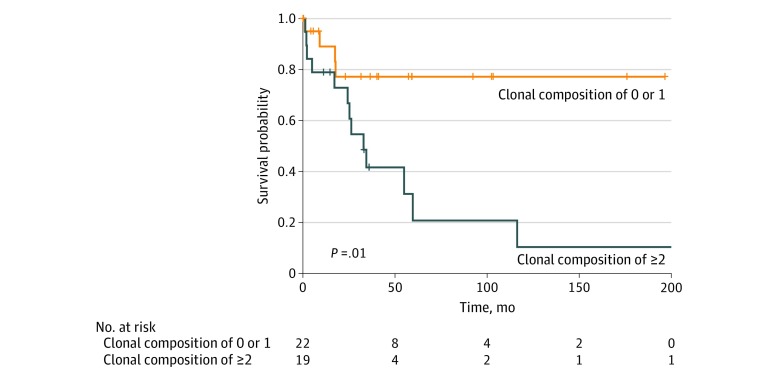
Kaplan-Meier Analysis of Survival in the Study Patient Population Data are stratified based on clonal composition count.

To examine the association of clinical outcome with increased heterogeneity, we stratified patients by clonal composition and performed survival analyses. Kaplan-Meier analysis demonstrated that cases with a clonal composition count of 2 or greater exhibited much worse survival compared with cases with a clonal composition count of 0 to 1 (univariate hazard ratio, 3.92; 95% CI, 1.27-12.08; *P* = .02) (Figure 1). This observation remained significant on multivariate analysis controlling for stage (T4 vs other), Lauren histologic subtype (intestinal vs other), receipt of adjuvant therapy (yes vs no), and age (continuous) (multivariate hazard ratio, 4.55; 95% CI, 1.09-19.04; *P* = .04). This finding aligns well with pancancer analyses of exome-sequencing data in which bioinformatic approaches have associated worse survival with higher intratumoral clone numbers in other tumor types.^7^

### Confirmation of Intratumoral Heterogeneity and Ascertainment of Spatial Relationships by Multiprobe FISH

**Figure 2.**
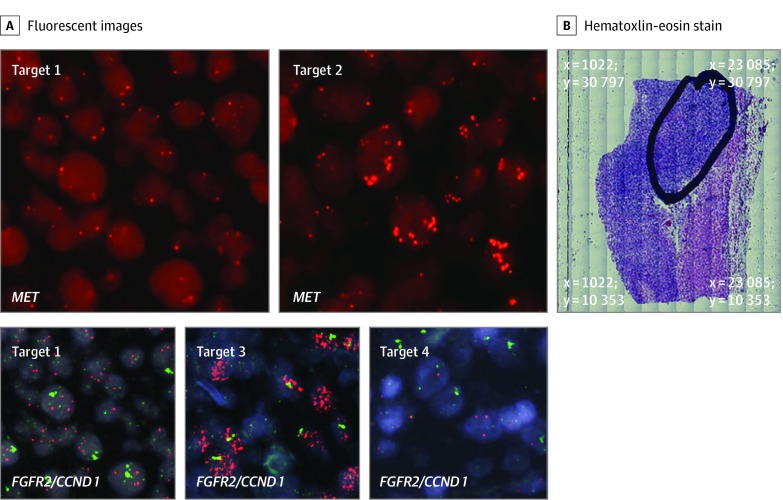
Patient 29 The patient had a pT3N0 Lauren diffuse subtype adenocarcinoma arising from the gastroesophageal junction with copy number alterations in *MET, FGFR2, CCND1, EGFR, CD274*, and *PDCD1LG2*. A, Target 1 images reside at coordinates x = 14 124 μm and y = 23 706 μm and exhibited 3 copies of *MET*, amplified *CCND1*, and 3 to 4 copies of *FGFR2*. Target 2 image resides at coordinates x = 15 145 μm and y = 23 424 μm and exhibited amplified *MET*. Based on OncoScan data reporting *FGFR2* amplification, additional targets were captured after initial analysis. An area of highly amplified *FGFR2* was subsequently identified in target 3 (x = 13 505 μm and y = 23 449 μm). Target 4 resides the greatest distance from target 1 at coordinates x = 11 002 μm and y = 27 428 μm and exhibited a normal copy number for *CCND1* and *FGFR2*. B, The tumor area of interest is circled on the hematoxylin-eosin–stained (H&E) slide section with the x, y reference coordinates at the 4 corners of the image displayed in micrometer distances. All fluorescent images were obtained at magnification x60; whole H&E tumor slide section image, magnification x5.

With evidence of significant intratumoral heterogeneity determined by the OncoScan SNV array calculation of clonal composition, we pursued multiprobe FISH analyses to ascertain whether detected multiple CNAs were restrained to a single cell or distributed among differing clonal populations. We initially focused on patient 29, who had a pT3N0 diffuse subtype adenocarcinoma arising from the gastroesophageal junction with amplification of *MET*, *FGFR2*, and *CCND1* (eFigure 4A in the Supplement). Copy number gains in *EGFR* and copy number loss in *CD274* and *PDCD1LG2*, which encode programmed death ligands 1 and 2 (PD-L1 and PD-L2), respectively, were also detected. Analysis of clonal composition had determined the existence of 3 clones, although this may be an underestimate owing to increased ploidy of the sample. In-depth multiprobe FISH analysis revealed intratumoral heterogeneity for *MET* gene amplification and heterogeneity in coamplification of other oncogenes among differing spatial regions. In the primary tumor region (designated target 1), tumor cells exhibited only 3 copies of *MET*, 4 to 7 copies of *EGFR,* 3 to 4 copies of *FGFR2,* single copies of the genes encoding PD-L1/PD-L2, and significant amplification of *CCND1* (Figure 2 and eFigure 5 in the Supplement). Target 2, which resided 1.06 mm from target 1, exhibited tumor cells containing both strongly amplified *MET* and *CCND1*, 3 to 4 copies of *EGFR*, 3 to 4 copies of *FGFR2*, and loss of both copies of the genes encoding PD-L1/PD-L2. To localize the strong *FGFR2* amplification reported by OncoScan but not exhibited in targets 1 or 2, we scanned the tumor area finding target 3, which resided 0.67 mm from target 1 and exhibited strong FISH amplification of *FGFR2* along with *CCND1*. To further confirm whether other intratumoral areas may be heterogeneous for *CCND1* amplification, we identified target 4, which resided 4.86 mm away from target 1, in which tumor cells harbored only 2 copies of *CCND1* and 2 copies of *FGFR2*. Thus, in total for this case we observed 4 differing FISH oncogene coamplification patterns exemplifying significant spatial intratumoral heterogeneity (Figure 2).

**Figure 3.**
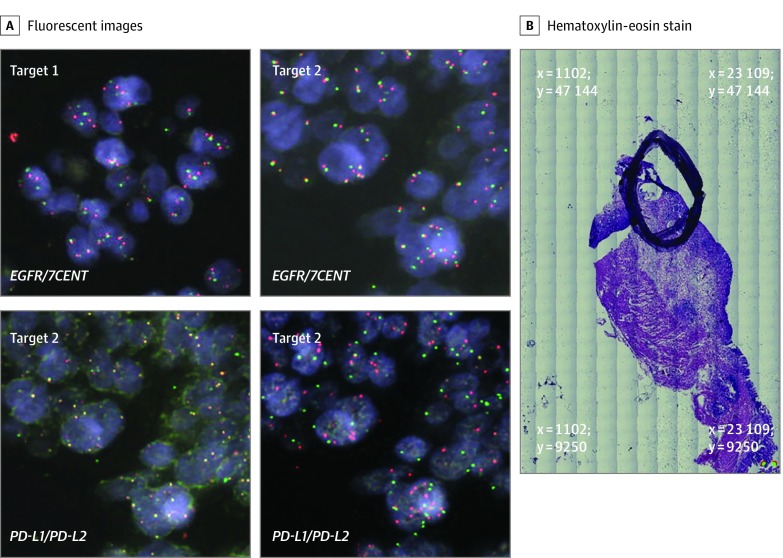
Patient 37 This patient had a pT2N3a Lauren intestinal subtype adenocarcinoma arising from the gastric cardia. OncoScan analysis revealed major copy number alterations of *CD274, PDCD1LG2, EGFR, MET*, and *PIK3CA.* A, Target 1 image at coordinates x = 13 654 μm and y = 33 594 μm exhibited modest copy number gains of *EGFR*. Target 2 images at coordinates x = 11 292 μm and y = 34 466 μm exhibited amplification in all 5 oncogenes. B, The tumor area of interest is circled on the hematoxylin-eosin–stained (H&E) slide section with the x, y reference coordinates at the 4 corners of the image displayed in micrometer distances. All fluorescent images were obtained at magnification x60, and whole H&E tumor slide section image, magnification x5.

The complementary role of multiprobe FISH was confirmed in patient 37, with a pT2N3a intestinal subtype adenocarcinoma arising from the cardia. OncoScan analysis revealed gain of the genes encoding PD-L1 and PD-L2 and *EGFR*, *MET*, and *PIK3CA* (eFigure 4B in the Supplement). Testing for Epstein-Barr virus was also pursued, with no evidence of infection by viral RNA in situ hybridization. Clonal composition analysis had determined the existence of 3 clones. In depth multiprobe FISH analysis observed 2 major spatial regions spaced 2.52 mm apart in the tumor that exhibited variability in low and high copy gains of multiple oncogenes (Figure 3 and eFigure 6 in the Supplement). Target 1 demonstrated amplification of the genes encoding PD-L1 and PD-L2 but only modest copy gains in *EGFR, MET*, and *PIK3CA*. However, target 2 exhibited amplification of all 5 genes of interest. As such, in this sample we observed 2 FISH coamplification patterns in which the genetic events for PD-L1 and PD-L2 appear to be truncal alterations common to both target regions. However, amplification of *EGFR*, *MET*, and *PIK3CA* appear to be subclonal, in which all 3 oncogenes are coamplified within the same tumor cells as opposed to being each individually amplified across mutually exclusive clonal populations.

**Figure 4.**
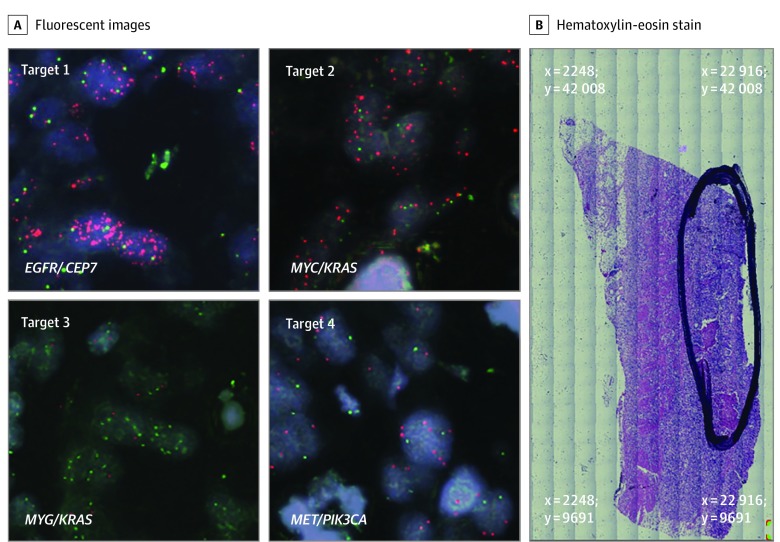
Patient 21 This patient had a pT3N0 Lauren intestinal subtype adenocarcinoma arising from the gastric antrum. OncoScan analysis reported amplification of *EGFR*, *MYC*, *KRAS, MET*, and *PIK3CA.* A, Target 1 image resides at coordinates x = 16 854 μm and y = 24 633 μm and exhibited amplified *EGFR*. Target 2 image resides at coordinates x = 17 387 μm and y = 25 310 μm and demonstrated amplified *MYC* with normal *KRAS* copy number. Target 3 image resides at coordinates x = 17 682 μm and y = 25 188 μm and exhibited the converse of target 2 with amplified *KRAS* but normal *MYC* copy number. Target 4 image resides at coordinates x = 17 407 μm and y = 24 485 μm and demonstrated amplified *MET* with normal copy number for *PIK3CA*. B, The tumor area of interest is circled on the hematoxylin-eosin–stained (H&E) slide section with the x, y reference coordinates at the 4 corners of the image displayed in micrometer distances. All fluorescent images were obtained at 60 × magnification; whole H&E tumor slide section image, 5 × magnification.

Finally, we focused on patient 21, with a pT3N0 intestinal histologic subtype tumor arising from the gastric antrum characterized by gains of *EGFR*, *MYC* (OMIM 190080) *KRAS*, *MET*, and *PIK3CA* by OncoScan (eFigure 4C in the Supplement). Clonal composition analysis had determined the existence of 2 clones. Target area 1 exhibited amplification in *EGFR* only with normal copy numbers of *MET*, *MYC*, and *PIK3CA* and modest copy number gain of *KRAS* (Figure 4 and eFigure 7 in the Supplement). Target 2 demonstrated amplification of *MYC* but normal copy numbers of *KRAS* as well as *EGFR*, *MET*, and *PIK3CA*. Target 3 exhibited amplification of *KRAS* only, but a normal copy number of *MYC*. Target 4 demonstrated amplification of *MET*, but a normal copy number of *PIK3CA*. In terms of intratumoral distances from target 1, target 2 resided at 0.86 mm, target 3 resided at 0.99 mm, and target 4 resided at 0.57 mm. As such, although OncoScan analysis of the entire tumor section reported coamplification of multiple oncogenes, in-depth multiprobe FISH analysis discerned that differing subclonal tumor cell populations each contain amplification of a mutually exclusive single oncogene.

## Discussion

The inherent spatial intratumoral heterogeneity of oncogenic CNAs present in de novo disease illustrates the challenges in developing targeted GEA therapies. In our data set, we observed nearly one-half (46%) of the cases exhibiting significant subclones detected via SNV array analysis and computational tools to estimate the number of clonal populations. Our spatial FISH analyses also exemplify intercellular genomic heterogeneity of actionable oncogenes at distances of less than 1 mm and greater than 4 mm. Detection of heterogeneity across large intratumoral distances can be abrogated with multiregion sampling of a tumor. However, determination of heterogeneity at submillimeter distances will likely require single-cell resolution as demonstrated herein to offer insight into strategies on combinatorial and/or sequential targeted and immunotherapeutic approaches. Variation in CNAs within differing clonal populations adds to the literature of caution needed in informing clinical treatment decisions based on next-generation sequencing analyses of small biopsy samples that pool tumor DNA, invariably representing metagenomes of multiple clonal populations.^7^ Our present data set supports in-depth analysis of the spatial intratumoral landscape at de novo GEA presentation to provide a road map for initial clonal composition. The development of intratumoral heterogeneity has been attributed to the gradual accumulation of mutations over time selecting for favorably growing subclones.^15,16,17^ Other reports also support a single catastrophic genome-wide mutational and chromosomal rearrangement event (chromothripsis), which subsequently drives the outgrowth of a selectively favorable clone.^18,19,20^ In our data set via OncoScan analysis, we observed an example of a chromothripsis event having occurred during tumor evolution. An improved understanding of mechanisms driving heterogeneity will be key to developing therapeutic strategies in all stages of GEA because appreciation of factors influencing innate and acquired drug resistance is necessary to optimize outcomes.

Kwak et al^3^ reported patient cases of *MET* and *ERBB2* coamplification occurring in the same tumor cells, including a single case of *MET*, *ERBB2*, and *EGFR* coamplification all occurring within a single tumor cell population. We also observed these findings, but in addition demonstrated cases at initial diagnosis in whom the primary tumor contains heterogeneity in which only a single oncogene is amplified in some tumor cells, and other tumor cells contain amplification of a differing oncogene. This finding hypothetically can affect targeted therapy strategies in which combination therapy is likely needed, in cases of coamplification within the same tumor cell, compared with sequential targeted therapy potentially used to eliminate successive clones in cases of coamplification occurring among heterogeneous tumor cell populations. Circulating tumor DNA may also have limitations in distinguishing these 2 mechanisms of coamplification, and as such repeated tumor biopsies may still need to be considered to provide spatial heterogeneity and enhance the temporal heterogeneity demonstrated by liquid biopsy. One may be able to infer through single-cell analyses that if only single oncogene amplification is captured, then identification of coamplification in circulating tumor DNA is likely accounted for by another clonal population residing in a nonsampled site. Thus, biopsy of at least a single metastatic lesion complemented by circulating tumor DNA analysis may provide a sufficient composite of tumoral heterogeneity at a given point in a patient’s therapy and avoid the invasiveness of sampling every metastatic site. Although limited by sample size, the immediate clinical implications of heterogeneity assessment are highlighted by our survival analyses, which suggest that more heterogeneous tumors carry a worse prognosis.

### Limitations

Weaknesses of our study include the retrospective nature of the analysis without pairing of our genomic findings to clinical response data from targeted therapeutics. However, the restriction of our sample selection to cases treated with up-front surgical resection avoids confounding the evolution of the tumor’s mutational profile that may arise under the pressure of antineoplastic therapies. Furthermore, no molecularly targeted agents as yet have a proven effect in the nonmetastatic setting for GEA that would have provided meaningful clinical annotation of response to such targeted strategies. Despite this limitation, our data further support the existence of significant intratumoral heterogeneity within untreated primary GEAs, and genomic analysis to the depth of single-cell approaches can be useful for cases in which surgical resection of the primary tumor has been performed. Understanding the spatial mapping of intratumoral heterogeneity will support ongoing efforts incorporating targeted therapeutics in the neoadjuvant setting for resectable disease. If such efforts fail, our demonstration of significant intratumoral heterogeneity of multiple oncogene coamplification events existing at nonmetastatic disease presentation may account for primary resistance to such approaches. Pectasides et al^1^ similarly observed heterogeneity in oncogene amplification within geographically distinct regions of the primary tumor and lymph node metastases, although such observations were limited to large intratumoral distances.^11^ As such, our finding of heterogeneity occurring at submillimeter distances in early-stage GEA is novel and should guide single-cell analytic efforts in refining genomic heterogeneity events.

## Conclusions

Our report represents, to our knowledge, one of the first efforts in GEA to demonstrate significant spatial intratumoral heterogeneity of relevant CNAs with clonal tumor cell populations coexisting at submillimeter distances. More heterogeneous tumors (clonal composition count of ≥2) exhibit worse clinical outcomes. Recognition of heterogeneous spatial coamplification patterns of intratumoral clonal populations existing at submillimeter distances lends support for single-cell analyses to guide precision medicine efforts.

## Notes

Supplement. **eFigure 1.** Distribution of Percentage of Genomic Changes **eFigure 2.** A Case With Significant Alterations in Whole-Genome Copy Number and B-Allele Frequency Plot **eFigure 3.** Oncoprint of CNAs and Mutations Detected With the OncoScan Platform Demonstrating Interpatient Tumoral Heterogeneity of Genomic Alterations of Selected Genes of Interest **eFigure 4.** OncoScan Genomic Data **eFigure 5.** Patient 29 Representing a pT3N0 Lauren Diffuse Subtype Adenocarcinoma Arising From the GEJ With CNAs in *MET, FGFR2, CCND1, EGFR, CD274*, and *PDCD1LG2* **eFigure 6.** Patient 37 Representing a pT2N3a Lauren Intestinal Subtype Adenocarcinoma Arising From the Gastric Cardia **eFigure 7.** Patient 21 Representing a pT3N0 Lauren Intestinal Subtype Adenocarcinoma Arising From the Gastric Antrum
