# Supplementary material for: Association Between Spatial Heterogeneity Within Nonmetastatic Gastroesophageal Adenocarcinomas and Survival
Source: JAMA Netw Open. 2020 Apr 27;3(4):e203652. doi: 10.1001/jamanetworkopen.2020.3652 (PMC7186861; doi:10.1001/jamanetworkopen.2020.3652)
Supplement: Supplement. — eFigure 1. Distribution of Percentage of Genomic Changes eFigure 2. A Case With Significant Alterations in Whole-Genome Copy Number and B-Allele Frequency Plot eFigure 3. Oncoprint of CNAs and Mutations Detected With the OncoScan Platform Demonstrating Interpatient Tumoral Heterogeneity of Genomic Alterations of Selected Genes of Interest eFigure 4. OncoScan Genomic Data eFigure 5. Patient 29 Representing a pT3N0 Lauren Diffuse Subtype Adenocarcinoma Arising From the GEJ With CNAs in MET, FGFR2, CCND1, EGFR, CD274, and PDCD1LG2 eFigure 6. Patient 37 Representing a pT2N3a Lauren Intestinal Subtype Adenocarcinoma Arising From the Gastric Cardia eFigure 7. Patient 21 Representing a pT3N0 Lauren Intestinal Subtype Adenocarcinoma Arising From the Gastric Antrum [file jamanetwopen-3-e203652-s001.pdf]

Supplementary Online Content

Chao J, Bedell V, Lee J, et al. Association between spatial heterogeneity within nonmetastatic gastroesophageal adenocarcinomas and survival. *JAMA Netw Open*. 2020;3(4):e203652. doi:10.1001/jamanetworkopen.2020.3652

eFigure 1. Distribution of Percentage of Genomic Changes

eFigure 2. A Case With Significant Alterations in Whole-Genome Copy Number and B-Allele Frequency Plot

eFigure 3. Oncoprint of CNAs and Mutations Detected With the OncoScan Platform Demonstrating Interpatient Tumoral Heterogeneity of Genomic Alterations of Selected Genes of Interest

eFigure 4. OncoScan Genomic Data

eFigure 5. Patient 29 Representing a pT3N0 Lauren Diffuse Subtype Adenocarcinoma Arising From the GEJ With CNAs in *MET, FGFR2, CCND1, EGFR, CD274*, and *PDCD1LG2*

eFigure 6. Patient 37 Representing a pT2N3a Lauren Intestinal Subtype Adenocarcinoma Arising From the Gastric Cardia

eFigure 7. Patient 21 Representing a pT3N0 Lauren Intestinal Subtype Adenocarcinoma Arising From the Gastric Antrum

This supplementary material has been provided by the authors to give readers additional information about their work.

©2020 Chao J et al. *JAMA Network Open*.

eFigure 1. Distribution of Percentage of Genomic Changes

A. Box plots representing distribution of percentage genomic changes between Lauren intestinal vs. diffuse subtype histology. Lines represent the median and 25th and 75th percentiles.

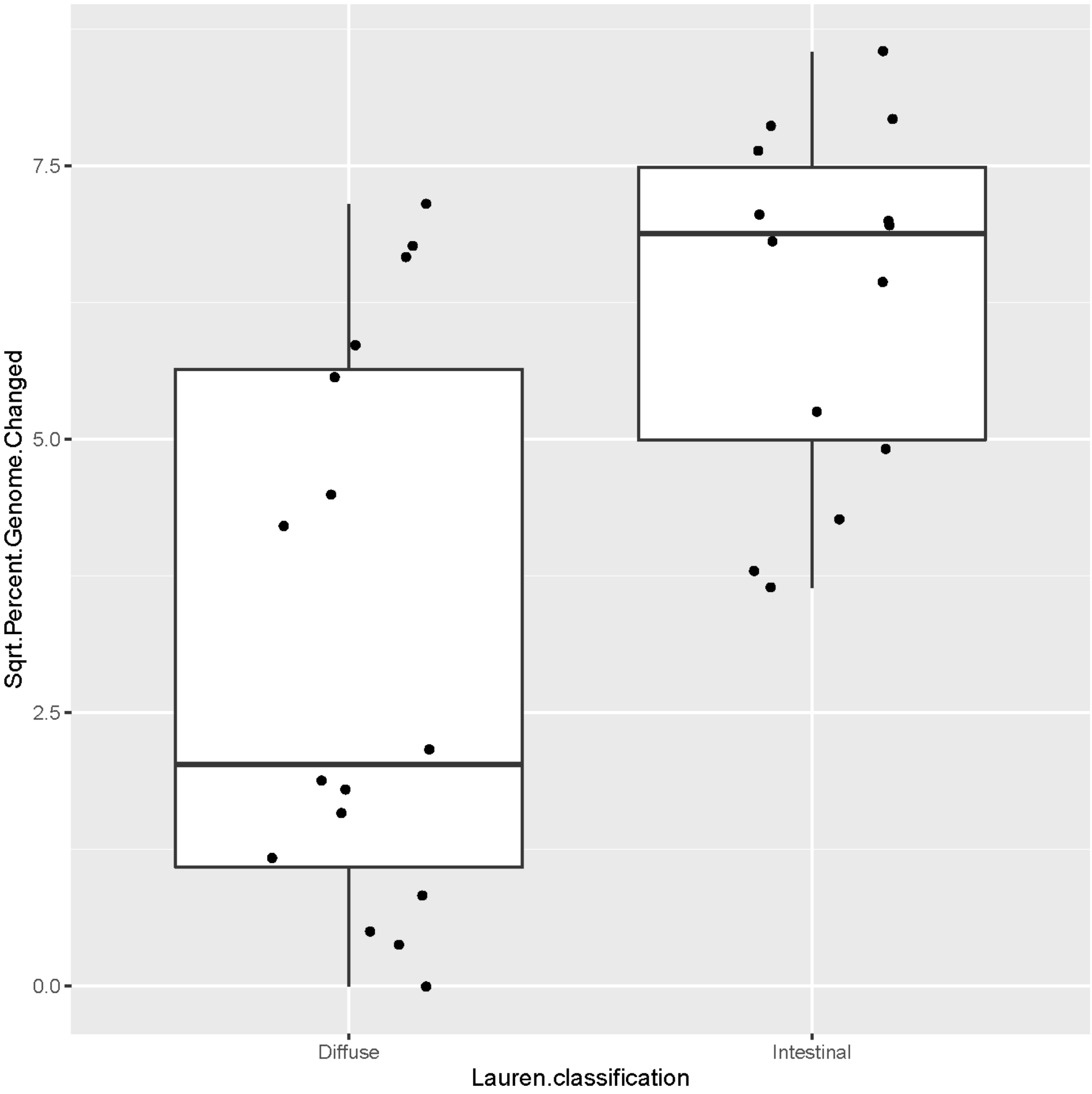

©2020 Chao J et al. *JAMA Network Open*.

B. Box plots representing distribution of percentage genomic changes between tumors arising from the GEJ/cardia/proximal stomach vs. Gastric body/antrum. Lines represent the median and 25th and 75th percentiles.

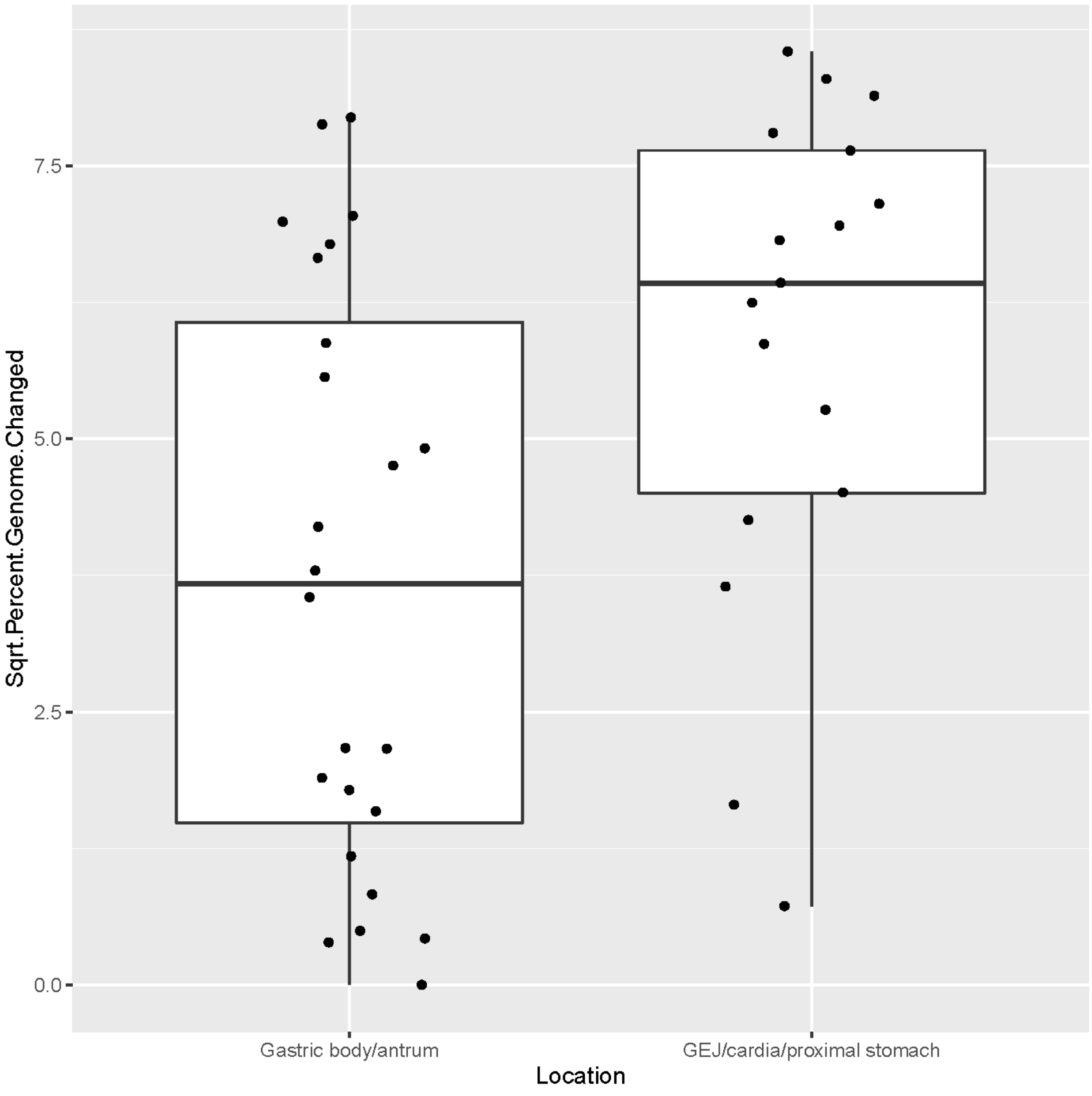

©2020 Chao J et al. *JAMA Network Open*.

eFigure 2. A Case With Significant Alterations in Whole-Genome Copy Number and B-Allele Frequency Plot

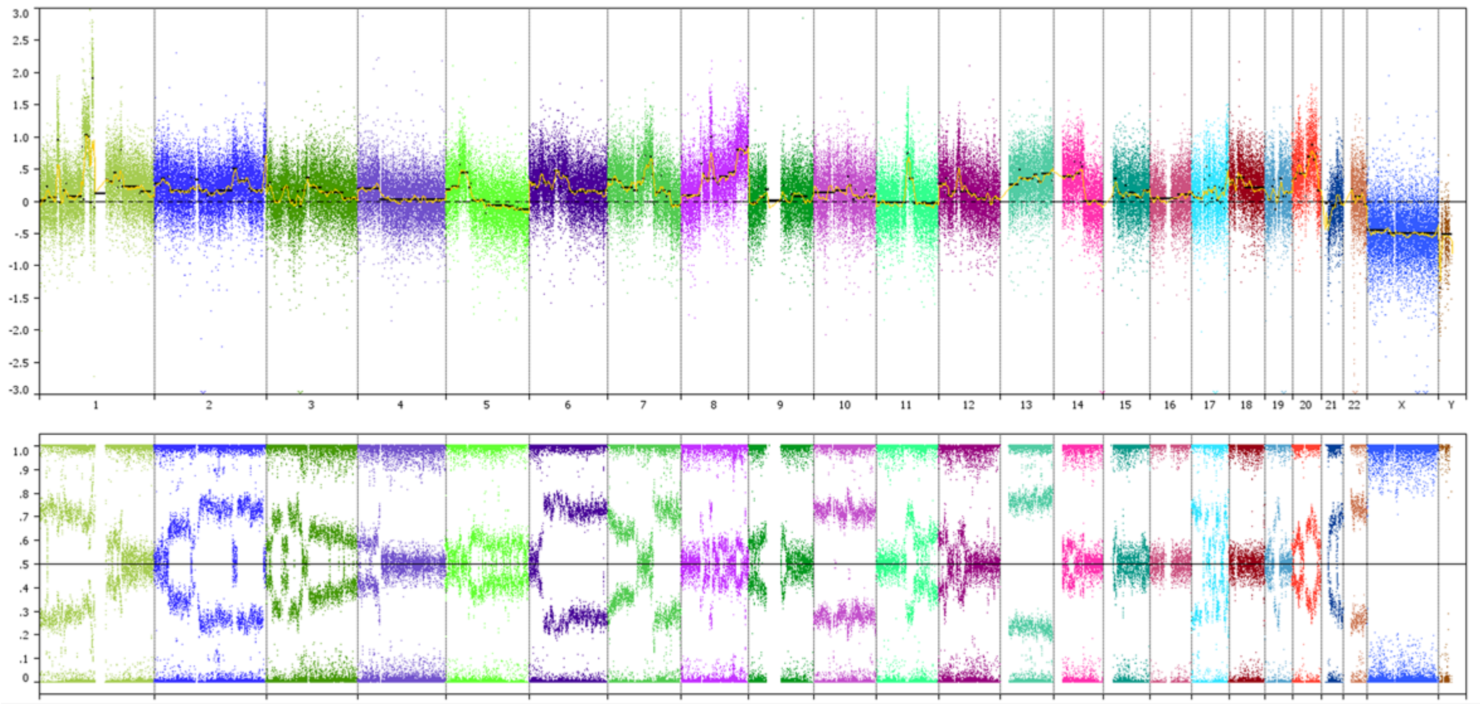

Top panel representing a case with significant alterations in whole genome copy number (y-axis, log2 scale) across multiple chromosomes (x-axis). The bottom panel represents the BAF (B allele frequency) plot (y-axis) in which multiple areas of the genome exhibit alternating retention and disruption of BAF representing both chromosomes (x-axis) with retention and loss of heterozygosity. This is indicative of chromothripsis being a major molecular event during this tumor's evolution.

©2020 Chao J et al. *JAMA Network Open*.

eFigure 3. Oncoprint of CNAs and Mutations Detected With the OncoScan Platform Demonstrating Interpatient Tumoral Heterogeneity of Genomic Alterations of Selected Genes of Interest
A variety of oncogenic CNAs were observed across patients including high copy gains in *EGFR, JAK2, FGFR2, MET, VEGFA, KRAS, NRAS, PIK3CA, CCNE1, CCND1, CDK4, CDK6,* and *AURKA*. One sample exhibited co-amplification of *CD274* and *PDCD1LG2* (encoding PD-L1 and PD-L2), in addition to concurrent amplification of *ERBB2, JAK2, FGFR2, MET, KRAS, PIK3CA,* and *MDM2*.

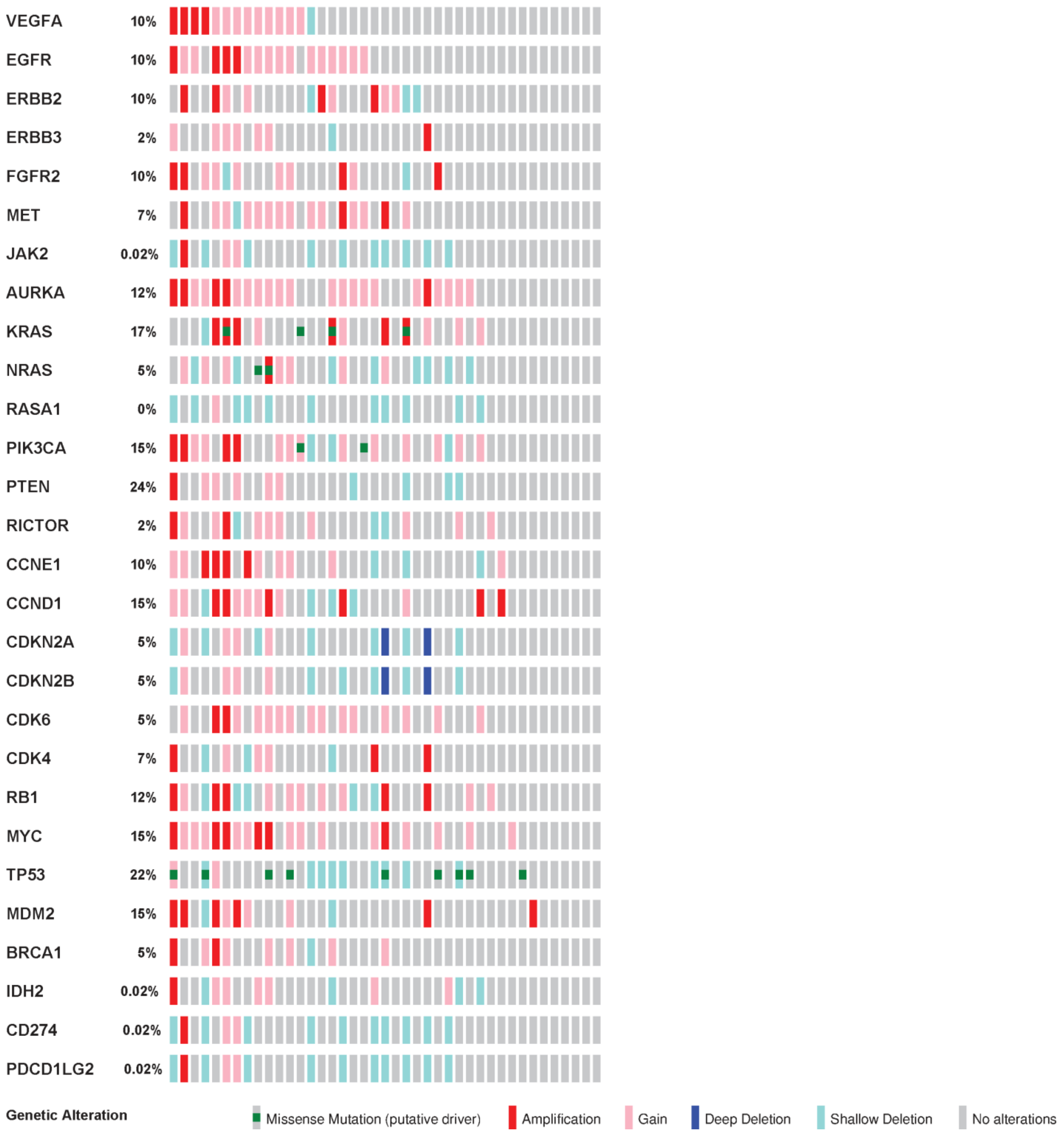

©2020 Chao J et al. *JAMA Network Open*.

eFigure 4. OncoScan Genomic Data

A. Oncoscan genomic data for Case 29.

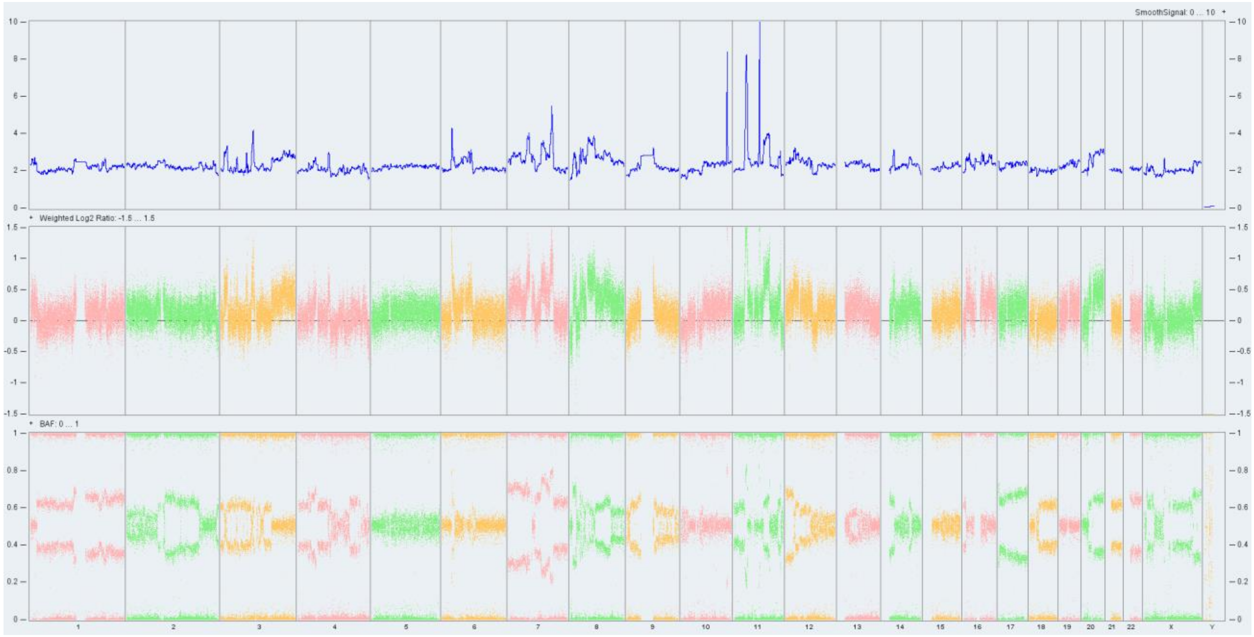

B. Oncoscan genomic data for Case 37.

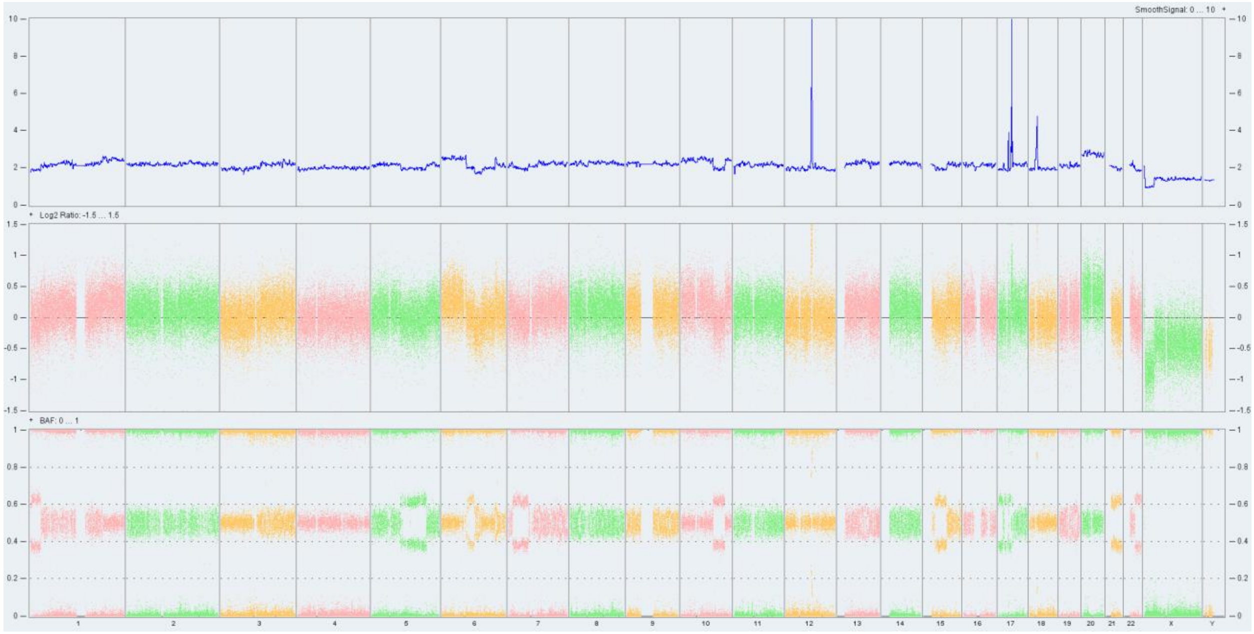

©2020 Chao J et al. *JAMA Network Open*.

C. Oncoscan genomic data for Case 21.

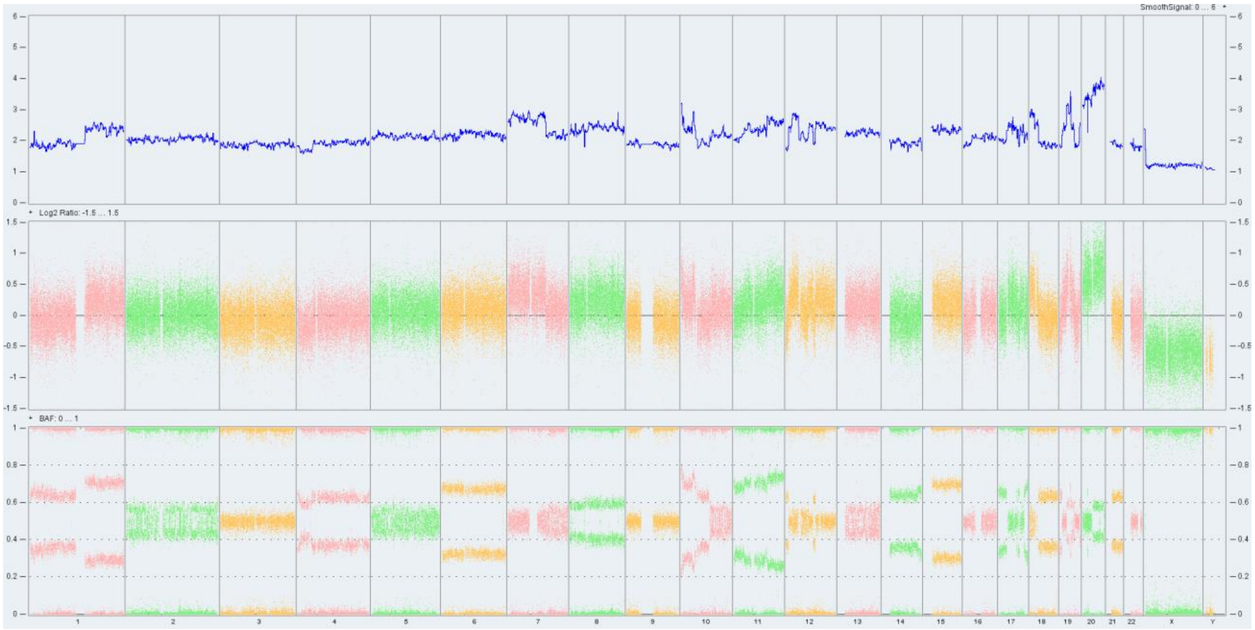

©2020 Chao J et al. *JAMA Network Open*.

eFigure 5. Patient 29 Representing a pT3N0 Lauren Diffuse Subtype Adenocarcinoma Arising From the GEJ With CNAs in *MET, FGFR2, CCND1, EGFR, CD274*, and *PDCD1LG2*

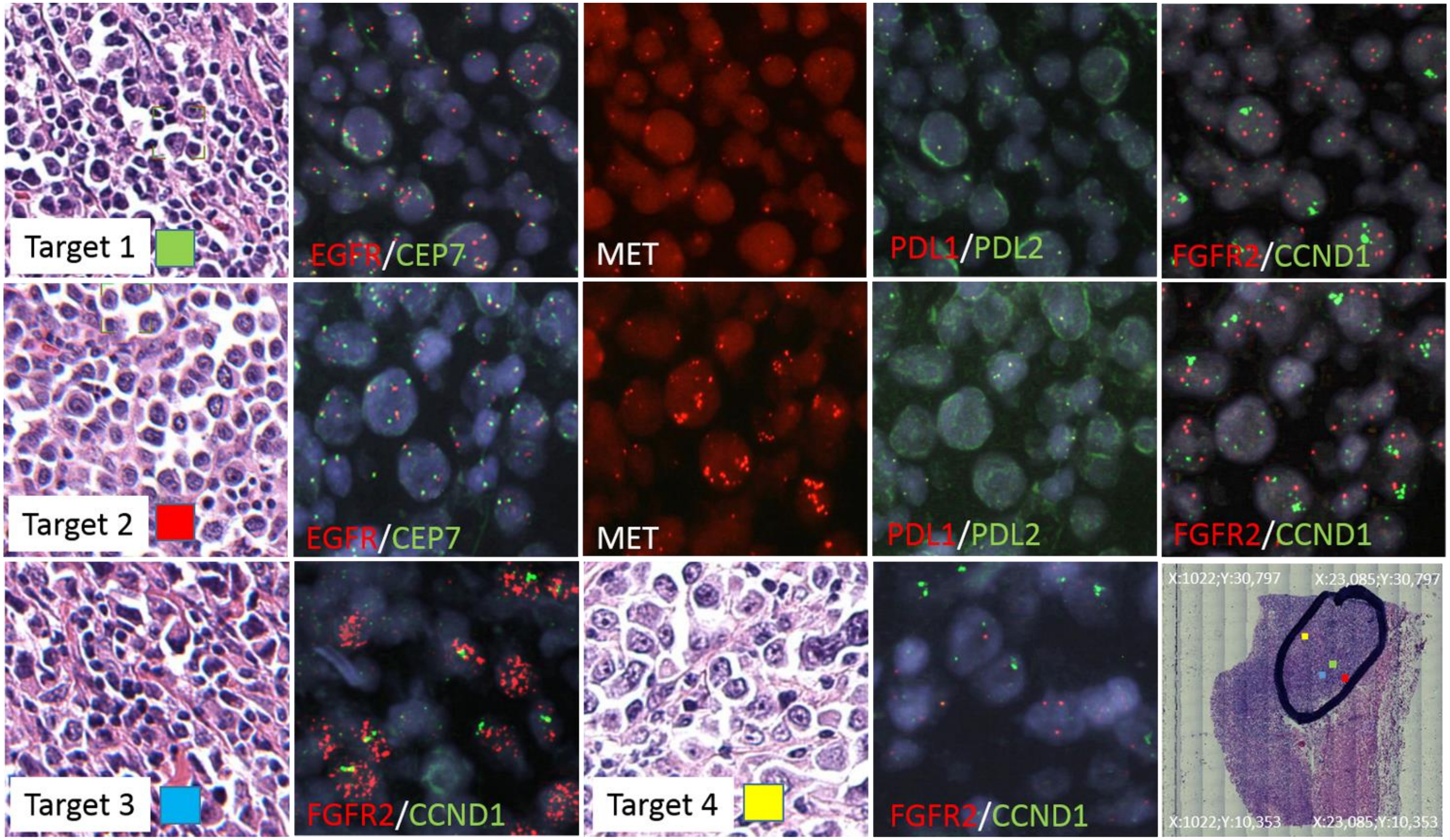

All fluorescent images are taken at 60X magnification, whole H&E tumor slide section image is at 5X, and zoomed in H&E is at 20X. The lower right panel is the tumor area of interest circled on the H&E slide section with the x,y reference coordinates at the four corners of the image displayed in micrometer distances. The top row encompasses images from Target 1 (green square) residing at coordinates x = 14,124 µm; y = 23,706 µm and exhibited 4-7 copies of *EGFR*, 3 copies of *MET*, 1 copy of the genes encoding PD-L1/PD-L2, amplified *CCND1*, and 3-4 copies of *FGFR2*. The middle row represents Target 2 (red square) residing at coordinates x = 15,145 µm; y = 23,424 µm and exhibited 3-4 copies *EGFR*, amplified *MET*, biallelic loss of the genes encoding PD-L1/PD-L2, amplified *CCND1* and

©2020 Chao J et al. *JAMA Network Open*.

3-4 copies of *FGFR2*. Based on Oncoscan data reporting FGFR2 amplification, additional targets were captured after initial analysis. An area of highly amplified *FGFR2* was subsequently identified in Target 3 (blue square, x = 13,505 µm; y = 23,449 µm). Target 4 (yellow square) resided the greatest distance from Target 1 at coordinates x = 11,002 µm; y = 27,428 µm and exhibited normal copy number for *CCND1* and *FGFR2*.

©2020 Chao J et al. *JAMA Network Open*.

eFigure 6. Patient 37 Representing a pT2N3a Lauren Intestinal Subtype Adenocarcinoma Arising From the Gastric Cardia

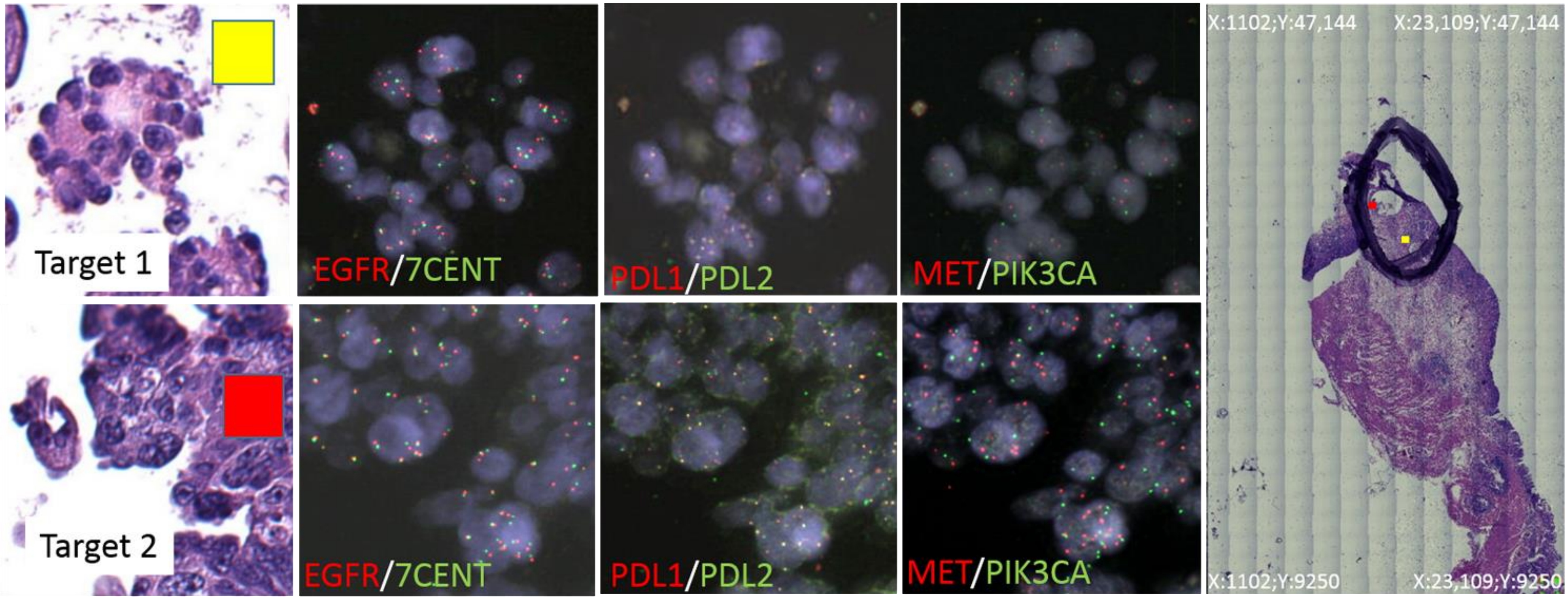

Oncoscan analysis revealed major CNAs of *CD274, PDCD1LG2, EGFR, MET*, and *PIK3CA.* Far right panel exhibits the tumor area of interest circled on the H&E slide section with the x,y reference coordinates at the four corners of the image displayed in micrometer distances. Top row encompasses Target 1 (yellow square) at coordinates x = 13,654 µm; y = 33,594 µm exhibiting amplification of the genes encoding PD-L1 and PD-L2, but modest copy number gains of *EGFR, MET,* and *PIK3CA*. Bottom row represents Target 2 (red square) at coordinates x = 11,292 µm; y = 34,466 µm exhibiting amplification in all 5 oncogenes. All fluorescent images are taken at 60X magnification, whole H&E tumor slide section image is at 5X, and zoomed in H&E is at 20X.

©2020 Chao J et al. *JAMA Network Open*.

eFigure 7. Patient 21 Representing a pT3N0 Lauren Intestinal Subtype Adenocarcinoma Arising From the Gastric Antrum

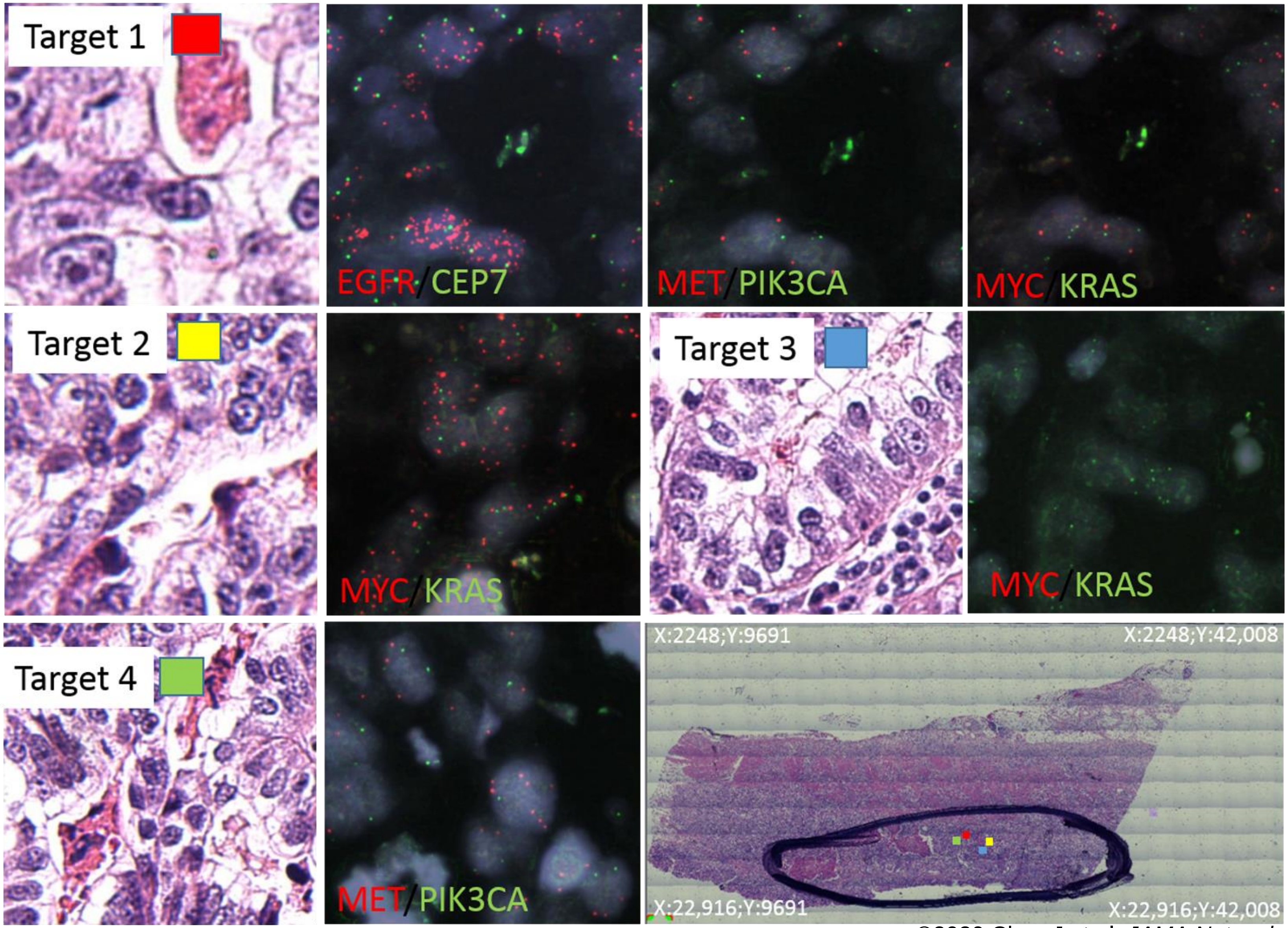

©2020 Chao J et al. *JAMA Network Open*.

Oncoscan analysis reported amplification of *EGFR, MYC, KRAS*, *MET* and *PIK3CA.* Bottom right panel exhibits the tumor area of interest circled on the H&E slide section with the x,y reference coordinates at the four corners of the image displayed in micrometer distances. Top row encompasses Target 1 (red square) at coordinates x = 16,854 µm; y = 24,633 µm and exhibited amplified *EGFR* with normal copy numbers of *MET, MYC*, and *PIK3CA* and modest copy number gain in *KRAS*. Target 2 (yellow square) resided at coordinates x = 17,387 µm; y = 25,310 µm and demonstrated amplified *MYC* with normal *KRAS* copy number. Target 3 (blue square) at coordinates x = 17,682 µm; y = 25,188 µm exhibited the converse of Target 2 with amplified *KRAS* but normal *MYC* copy number. Target 4 (green square) at coordinates x = 17,407 µm; y = 24,485 µm demonstrated amplified *MET* with normal copy number for *PIK3CA*. All fluorescent images are taken at 60X magnification, whole H&E tumor slide section image is at 5X, and zoomed in H&E is at 20X.

©2020 Chao J et al. *JAMA Network Open*.
